# Investigation of drag reduction by slurry-like drag-reducing agent in microtube flow using response surface methodology (RSM)

**DOI:** 10.1038/s41598-023-49804-9

**Published:** 2023-12-17

**Authors:** Zhensong Cheng, Xin Zhang, Xinwang Song, Xudong Wang, Guoxin Zhang, Yuan Lu, Lei Li, Feifan Liu, Xiaodong Dai

**Affiliations:** 1 College of Petroleum Engineering, Shandong Institute of Petroleum and Chemical Technology, Dongying, Shandong 257061 People’s Republic of China; 2 CNOOC (Tianjin) Oilfield Chemical Co., Ltd, Tianjin, People’s Republic of China

**Keywords:** Fluid dynamics, Mechanical engineering

## Abstract

In this study, we investigated the drag reduction property of a premixed slurry drag reducer in a millimeter-scale pipe. The aim of this study is to establish the correlation between Darcy friction factor and drag reducer concentration (*C*) and volume flow (*Q*). First, the experimental plan was designed by using the response surface method (RSM), and then the experimental data were processed to establish the quadratic correlation between the response variable and the description variable. After that, ANOVA analysis of variance was used to verify the accuracy of the experimental data and the correlation. Finally, the prediction model is extended to a larger concentration and volume flow range, and it is found that the accuracy between the predicted value of friction coefficient and the experimental value is ± 30%, thus verifying that the correlation is suitable for the small-scale fully turbulent region. Compared with traditional experimental design and correlation methods, the implementation of Response Surface Methodology (RSM) in this study not only reduces the experimental time but also yields a more robust correlation for predicting the friction coefficient.

## Introduction

Resistance in turbulence is a major issue in fluid dynamics due to its negative impact on the efficiency of fluid flow systems such as pipelines, ships, and aircraft^1–5^. In the past, researchers have attempted to reduce drag in turbulent flows via various techniques such as passive and active flow control. However, the most widely used technique is the addition of polymers to the fluid, which can significantly reduce drag in turbulent flow. Among these, the most frequently-used method is drag reducing polymer^6–8^.

Drag reducing polymer (*DRP*) is a high molecular weight polymer, which can reduce the frictional resistance between fluid (petroleum, diesel oil, etc.) and the inner wall of the pipeline, thereby reducing pump work and improving energy efficiency^9^. The earliest commercial application report of drag reducer originated in 1980s. Burger et al. applied polymer to the transportation of diesel and Sadlerochit crude oil in an oil pipeline with a length of 1278 km (48-inch inner diameter) across Alaska. The drag reduction rate was 21%^10^. Since then, BECKER has successfully applied drag reducer to crude oil transportation in alpine regions. At present, the technology of drag reduction by additives was widely used in the transportation of oil, gasoline and other fluids in Europe and Asia^11–15^.

Although there are numerous commercial applications of drag-reducing polymers, numerous unresolved issues remain that researchers are exploring. Park et al.^16^ developed a novel drag reducing self-polishing copolymer. In the measurement of skin friction, the polymer specimen exhibited a skin friction reduction ratio of 9.49% at the freestream flow speed 5 m/s. Virk^17^ summarized the experimental results of turbulent drag reduction of polymers in smooth-walled pipe flow, and divided the polymer action region into laminar flow region, polymer action region and asymptotic region of maximum drag reduction; and the maximum drag reduction asymptotic region, which provides a theoretical basis for subsequent research. Zakin et al.^18^ observed the change of the drag reduction rate by changing the concentration and molecular weight of *DRP* when the mixture of toluene and isooctane flowed in the turbulent region (1.58 mm inner diameter). The results showed that the drag reduction rate increased with the increase of the polymer concentration, when the number of Re was fixed. Martin et al.^19^ examined the variation of drag reduction rate by adding *DRP* with different molecular weights and concentrations to oil-soluble polyisobutylene solvent (30.2 mm inner diameter, length 0.648 m). The experiment proved that *DRP* has the maximum drag reduction rate at 70% of the maximum molecular weight. Alsurakji et al.^20^ evaluated the energy efficiency of polar water-soluble polyacrylamide and non-polar oil-soluble polyisobutylene in single-phase water and oil flow, two-phase air water and air oil flow, and three-phase air oil water flow. Ryskin et al.^21^ deduced an effective viscosity model through scalar analysis to simulate the effect of polymer stretching on the effective viscosity. In this model, the viscosity depends on the concentration of the polymer and the maximum stretching degree of the polymer molecule. Yang^22^ found that the drag reduction increased and then decreased with the increasing *Re* number for different polymer concentrations. Kim et al.^23^ used polyethylene oxide to determine the effect of polymer concentration and molecular weight on drag reduction. The results show that the drag reduction rate increases with the increase of molecular weight only at the concentration of 1 ppm, and this rule does not apply when it increases to 10 ppm. With increasing *Re* (from 30,000 to 60,000), the drag reduction rate increases overall (rather than continuously), but the trend is not fully experimentally verified. Quan et al.^24^ considered factors such as drag reducer polymer type, polymer concentration, Reynolds number, temperature and shear under turbulent flow, and obtained the effect of concentration, Reynolds number and shear time on drag reduction. The results showed that the temperature has a small effect on the drag reduction rate (inner diameter: 12.7 mm, length: 9.4 m). Zhang et al.^25^ studied the drag reduction law of dilute linear flexible polymer polyethylene oxide solution in fully turbulent pipe flow (inner diameter: 16 mm) through experiments, and concluded that the drag reduction rate is related to the Weissenberg number and polymer concentration. The experimental correlation formula ignores the influence of the dimensionless number *Re* on the drag reduction rate, which expands the application value of the drag reduction rate.

Previous works used regular-size tube or pipe to investigate the drag reduction, while, this paper analyzes experimental data of the friction factor in premixed slurry drag reducer solution system (an active drag reduction method) under same thermal conditions. Premixed slurry drag reducers are specialized chemical solutions or mixtures carefully designed to reduce the frictional resistance encountered by fluids as they flow through pipelines. These reducers are particularly important in industries such as oil and gas, mining, and water treatment. What makes premixed slurry drag reducers unique is their pre-blending with water or another suitable carrier fluid, which simplifies the process of directly applying them to the pipeline system. Unlike some other drag-reducing agents that require separate injection or addition to the fluid, premixed slurry drag reducers are already integrated with a carrier fluid. This inherent characteristic streamlines the application procedure, eliminating the need for additional equipment or mixing processes. Currently, few studies have attempted to use slurry-like drag reducer in the drag reduction work, so, in this study, the most innovative part is that we combine the RSM and slurry-like drag-reducing agent which is rarely used in the drag reduction experiment.

## Experimental setup and method

This microtube experiment uses a homogeneous experimental approach, in which polymers are first mixed in a solution before being pumped into the pipeline to measure the pressure difference^26^. The drag reducer solution was prepared using a concentrated slurry drag reducer and deionized water, mixed with a magnetic stirrer at low speed for 3 h until clusters or aggregates disappeared. Use it all in one day.

Concentrated slurry is a slurry-like substance composed of drag-reducing polymers, surfactants, and dispersants^27,28^. The drag-reducing polymer mainly serves to reduce the fluid viscosity and improve fluid flowability, while the surfactant can reduce the liquid surface tension and increase the contact area between solids and liquids, thereby enhancing the dispersion effect. The dispersant is a medium that disperses solids in liquids and can be water, oil, organic solvents, etc.

**Figure 1:**
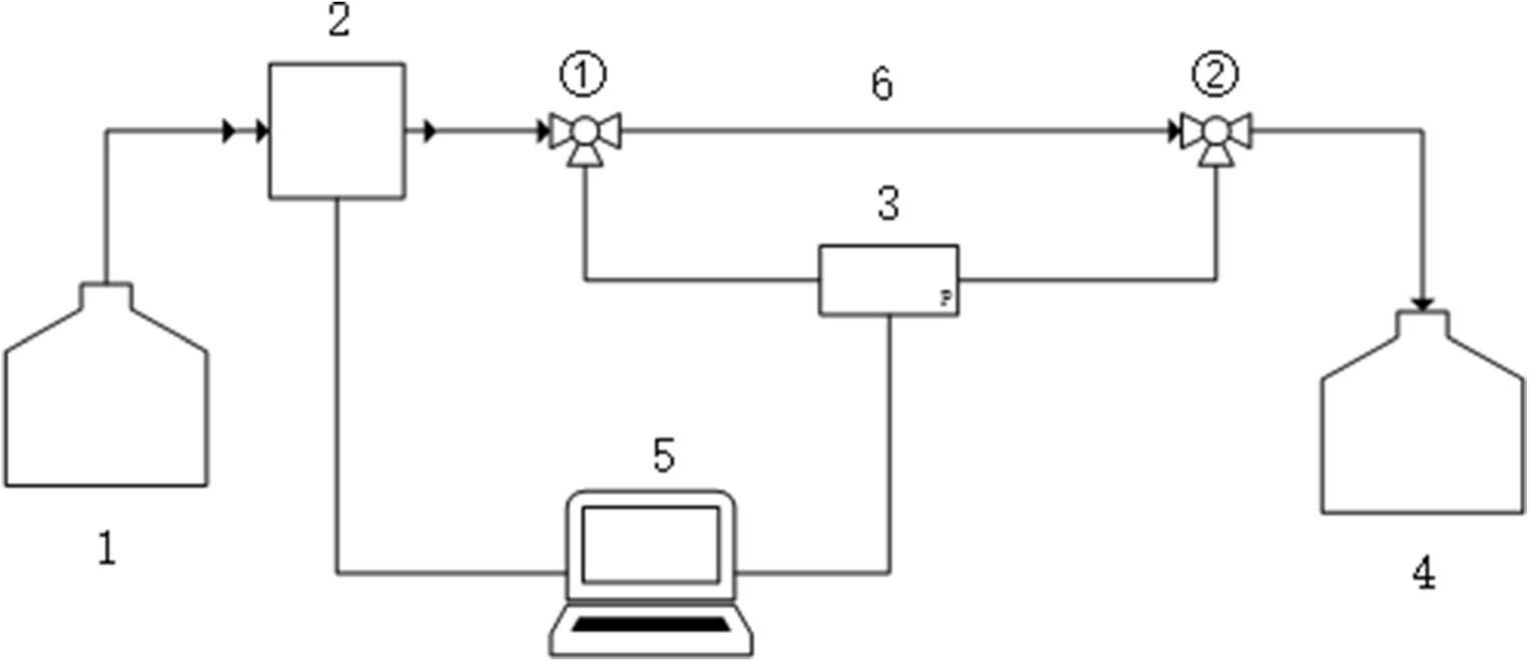
Flow diagram of fluid microflow drag reduction device. 1. Inlet solution storage device; 2. Advection pump; 3. Diaphragm Differential Pressure sensor; 4. Outlet solution storage device; 5. Computer; 6. Millimeter-scale microchannels.

The flow chart of the fluid microfluidic drag reduction device^29^ is shown in Fig. 1. The drag reducer solution was delivered to the test pipeline from a 2 L solution storage device by an advective pump. To eliminate the entrance effect of pipe flow, the entrance length of the test section is set to 0.4 m. The pressure differential sensor is used to measure the pressure difference between point 1 and 2 in the test section. In order to prevent the drag reducing agent from polluting the environment, the outlet is connected to the solution storage device. The test pipe adopts a transparent plastic round straight pipe. The inner diameter of the pipe test section is 1.85 mm and the length is 0.55 m. The maximum working pressure of the differential pressure gauge (EJA110E, YOKOGAWA) is 50 kPa, and the sampling period is set to 1 s.

To mitigate the influence of temperature on the variation of drag reduction rate, the drag-reducing agent solution was stabilized at 24 ± 0.5 °C before we start the experiment. The recording started when the Δ*p* data became stable. The corresponding *f* for the friction coefficient^25^ is given by

1 $$f = \frac{2d\Delta p}{{l\rho U_{b}^{ 2} }}$$

$\rho$ is the density of the solution, ${\text{kg}}/{\text{m}}^{3}$; *U*_*b*_ is the average velocity of the fluid section^25^ from Eq. (2), ${\text{m}}/{\text{s}}$. Based on previous experimental studies, it was found that the density of the solution did not change significantly after adding the drag reducing agent, which was not used as an experimental variable^30^.

2 $$U_{b} = \frac{Q}{{\pi r^{2} }}$$

*Q* is volume flows, ${\text{ml}}/{\text{min}}$.

According to Ebagninin et al.^31^, high concentration polymer solutions, such as PEO solutions, have a viscosity that is not significantly different from water. In this experiment, the maximum concentration is 100 ppm, which falls within the low concentration range. Therefore, the solvent viscosity is used to calculate the Reynolds number^26^ (Eq. 3) for result analysis under the current experimental conditions, and the viscosity at 24 °C corresponds to 0.9142 mPa s^32^. The viscosity table for water is required.

3 $${{Re}} = \frac{{\rho U_{b} D}}{\mu }$$

*D* is the inner diameter, m.

When the polymer is used as an additive in turbulent pipe flow, the drag reduction rate (*DR*)^1^ can be defined as the relative reduction of friction coefficient at the same Reynolds number:

4 $$DR = \frac{{f_{s} - f_{p} }}{{f_{s} }} \times 100\%$$

5 $$DR = f(d,U_{b} ,\mu_{s} ,C,N,M)$$

6 $$DR = f({{Re}} ,C)$$

where *f*_*s*_ is the Darcy friction coefficient without polymer; *f*_*p*_ is the Darcy friction coefficient of the polymer drag reducer solution under the same *Re*. In this definition, *DR* is a dimensionless number. At the same temperature, *DR* can be correlated with the experimental data by a polynomial fitting. So in this experiment, a specific polymer drag reducer was used, and the type and molecular weight of the polymer were not considered as variables. As mentioned earlier, the fluid viscosity was 0.9142 mPa s; by dimensionless average flow velocity, viscosity, and diameter into Reynolds number, the model can be simplified as a function of only Reynolds number and *C*, which can be represented by Eq. (6). The range flowrate we use in this work is from 50 to 400 ml/min. As shown in Table 1.

**Table 1:** Overlap Reynolds number (Re) at different volumetric flow rates.

*Q* (ml/min)	*U*_*b*_ (m/s)	*Re*
50	0.31	627.67
100	0.62	1255.35
150	0.93	1883.03
200	1.24	2510.71
250	1.55	3138.38
300	1.86	3766.06
350	2.17	4393.74
400	2.48	5021.42

**Figure 2:**
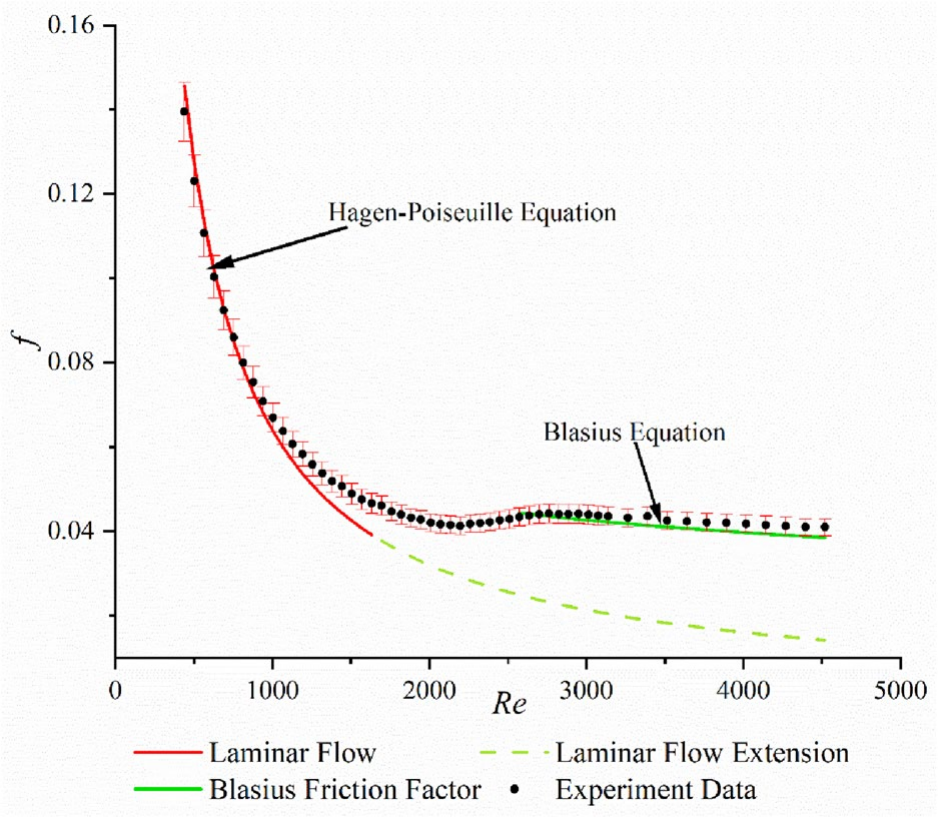
Darcy friction coefficient benchmark test for 0 ppm solution.

To verify the accuracy of the experimental equipment, a series of experiments were conducted using pure deionized water (without polymer). The measured Darcy friction factor was compared with the laminar Hagen–Poiseuille Eq. (7) and the classical turbulent Blasius Eq. (8)^25^, as shown in Fig. 2.

7 $$f = \frac{64}{{{Re}}}$$

8 $$f = 0.3164{{Re}}^{ - 0.25}$$

The classical friction relationships for fluid flow through flow passages are different for the various flow regimes and are typically distinguished by the Reynolds number. For turbulent flow with 4000 < *Re* < ${10}^{5}$, a simple relation is the so-called Blasius equation (Eq. 8). For *Re* < 2300, or laminar flow, the friction factor *f* depends only on Reynolds number and is given by Hagen–Poiseuille equation (Eq. 7). The reason why the transitional Reynolds number is different from the one in classic theory due to the transport of fluid in microtube. In microtube flow, the is small, so shear rate is high under the same flow rate or velocity. Thus, the transport in microtube is fierce and cannot be predicted by the classic theory. So, the transitional Reynolds number is not the same as the classic theory^33^.

In this figure, the error bar is set at 5% for each friction factor. When we combine the error bar with the data, we can see that the experimental results agree with theoretical values, which are calculated by the Hagen–Poiseuille equation and the Blasius equation. Thus, it is seen that the friction factor measured in the experiments is very close to the theoretical one. We can further ensure that all measurements are correct and reasonable. Since our device can provide correct data, there is no need to add error bars for all data.

Based on the Fig. 2, it is evident that the measured friction factor (*f*) of the base liquid (water) gradually decreases as the Reynolds number increases within the laminar flow region. Besides, the friction factors measured in laminar flow follow the one predicted by Hagen–Poiseuille equation for laminar flow when the Reynolds number is less than 1000. In the turbulent flow development region (*Re* > 2800), the friction factor *f* slowly decreases with the increase of Reynolds number, it follows the Blasius equation. In these two flow regimes, the friction factor can be predicted by two classic equations, so we can confirm that experimental data measured in our experimental device are accurate. In the transitional region (Reynolds number greater than 1000 and less than 2800), the friction factor cannot be predicted by the two classic equations mentioned above, due to the complex flow regime^34^.

The relevant Reynolds number range of the two formulae is not entirely compatible with the experimental results, as can be seen from the analysis above. Ghajar et al. also observed similar results^35,36^, which may be related to the data variations brought on by the test pipes and equipment.

**Figure 3:**
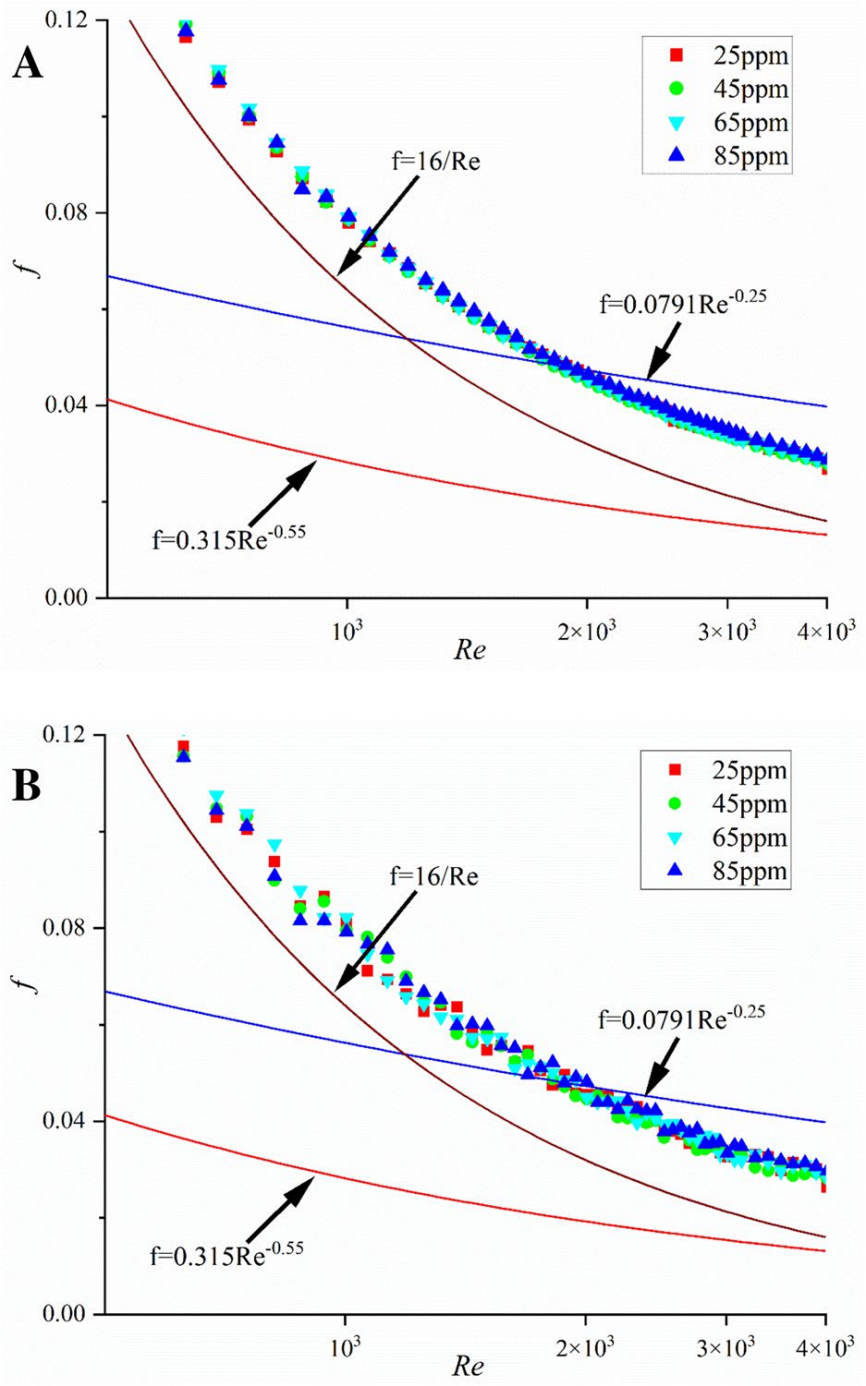
The relationship between friction factor and Reynolds number at different concentrations. (**A**) and (**B**) depict two sets of experimental data, serving to validate the reproducibility of the results (blue line: Blasius friction factor scaling; brown line: Hagen–Poiseuille flow; red line: Zakin’s asymptote).

In Fig. 3, the measured Darcy friction factor is presented at various Reynolds numbers for the drag-reducing flow. To investigate the repeatability and reproducibility of experimental results, we conducted additional experiments to assess the consistency of the findings. The data in Fig. 3A,B exhibit are almost the same, and we confirm that the reparability of our data is good. As previously explained, we calculate the Reynolds number using the solvent viscosity, since, in dilute polymer solutions, the presence of polymers does not substantially alter viscosity. In the turbulent zone characterized by Reynolds numbers (*Re*) exceeding 2000, several noteworthy observations come to the forefront. Firstly, it is worth noting that the friction coefficient associated with the polymer consistently registers values lower than those predicted by the Blasius equation. This discrepancy strongly implies the absence of drag reduction in this specific turbulent regime. Also, the friction factors of flow are higher than the friction factors predicted by Zakin’s asymptote^37^, Eq. (9), which represents the minimum friction factor by surfactant.

**Figure 4:**
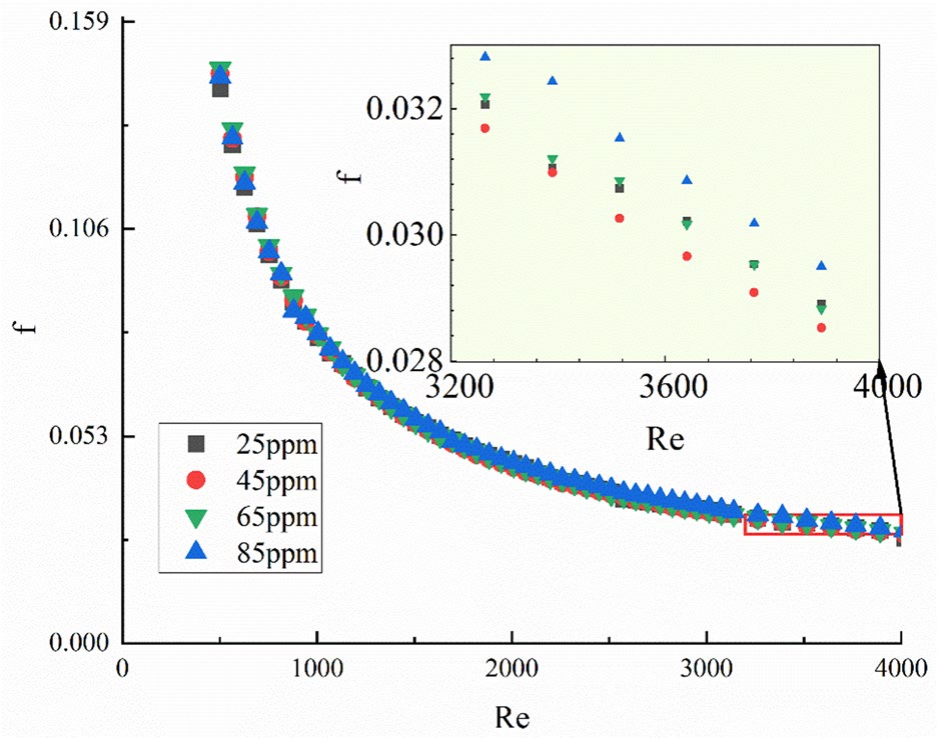
Relationship of *Re* and *f* (subfigure for clarification of drag reduction under some concentrations).

The qualitative analyses conducted in this study serve to bolster the validity of the experimental data and provide insights into the influence of concentration on drag reduction. As illustrated in Fig. 4, the relationship between drag reduction and polymer concentration reveals a discernible pattern characterized by an initial decrease followed by an increase in the overall friction factor as the concentration increases. At optimal concentrations, polymers exhibit an enhanced ability to suppress turbulent structures, facilitating the efficient utilization of energy dissipated by these turbulent structures for upward flow. However, when the concentration exceeds this optimal range, there is no significant improvement in the friction factor. It is conceivable that at concentrations surpassing a critical threshold, the drag-reducing agent may excessively aggregate or deposit on fluid or solid surfaces, resulting in uneven distribution^38,39^. This non-uniform distribution could lead to variations in frictional resistance, thereby elevating the friction factor.

9 $$f = 0.315{{Re}}^{ - 0.55}$$

## Results and discussions

Response Surface Methodology (RSM) is an experimental design and optimization technique developed in the 1950s^40^. RSM is a powerful tool for designing experiments, developing models, and optimizing processes or systems by analyzing the relationship between predictor variables and the response variable. It involves creating response surface models to optimize reaction conditions and maximize or minimize the response variable. By fitting experimental data, a global regression equation is obtained, and optimal factor levels are determined.

RSM uses two additional coefficients to calculate the statistics of experimental data, namely adjusted ${{R}}^{2}$ and predicted ${{R}}^{2}$, which have proven to be advantageous. For example, in the traditional method, a larger ${{R}}^{2}$ can be produced by using more data points (more variables and responses) even though the correlation does not fit the data well. This can create difficulty in evaluating the accuracy of a correlation. The use of adjusted ${{R}}^{2}$ in RSM can solve this problem by excluding the effects of a large number of variables and responses. A larger adjusted ${{R}}^{2}$ indicates a good fit of the data to the developed correlation. Moreover, RSM uses predicted ${{R}}^{2}$, which is calculated from a portion of the entire dataset, to predict other observations in the remaining data. Unlike ${{R}}^{2}$, which is always a positive number, adjusted ${{R}}^{2}$ and predicted ${{R}}^{2}$ can be positive or negative. When either of them is negative, the correlation format should be modified to better fit the data.

With the RSM, a quadratic formulation is used to correlate experimental data.

10 $$Y = \beta_{0} + \sum {\beta_{i} X_{i} } + \sum {\beta_{i} X_{i}^{2} } + \sum {\beta_{ij} X_{i} X_{j} }$$

The experimental response variable ${{Y}}$ is the friction factor *f*; the actual factors ${{X}}_{{i}}$ and ${{X}}_{{j}}$ are the volume flow *Q* and the drag reducing agent concentration *C* respectively; $\beta_{0}$ is a constant term, $\beta_{{i}}$, $\beta_{{j}}$ and $\beta_{{ij}}$ are linear terms, quadratic term and interaction term coefficients (i = 1,2; j = 1,2).

The RSM is based on a Completely Randomized Design (CRD), and the order in the table was randomly generated by software (Design expert 13, free-trail version). The CCD (Central Composite Design) of RSM requires that the center point of each factor be included in order to complete the design and calculation. According to the RSM experimental design method, only 13 experimental points are needed, and the measurement values of f are summarized in Tables 2 and 3.

**Table 2:** Experiment design.

Factor	Lowest level (− 1)	Median (0)	Highest level (+1)
Volume flow *Q* (ml/min)	220	260	300
Concentration *C* (ppm)	35	45	55

**Table 3:** Experiment values of *f* with RSM.

Concentration (ppm)	Volume flow (ml/min)	Darcy friction factor
35	300	0.029
45	260	0.032
55	220	0.037
55	260	0.033
45	300	0.029
35	220	0.036
45	220	0.036
45	260	0.032
45	260	0.032
45	260	0.032
45	260	0.032
35	260	0.032
55	300	0.030

Bringing the experimental data in Table 3 into the RSM processing model, a correlation in Eq. (11) can be obtained.

11 $$f = 2.24 \times 10^{ - 2} - 1.06 \times 10^{ - 4} C - 7.2 \times 10^{ - 5} Q + 5.21 \times 10^{ - 8} CQ + 1.10 \times 10^{ - 6} C^{2} + 9.36 \times 10^{ - 8} Q^{2}$$

To verify the accuracy of the correlation equation, ANOVA variance analysis method was used, and detailed data can be found in Table 4. It can be seen from the table that the total ${{P}}$ value of the model is less than 0.0001. The correlation fitting statistical Table 5 shows that the adjusted ${{R}}^{2}$ and adjusted ${{R}}^{2}$ are 0.999 and 0.998, respectively. The results in Tables 4 and 5 indicate a good correlation between the experimental data and Eq. (11). These results indicate that the correlation presented in Eq. (11) has a good accuracy in predicting the measured ${{f}}$.

**Table 4:** ANOVA table for simplification.

Source	Sum of squares	*df*	Mean square	*F*-value	*P*-value
Model	0.0001	5	0.0000	1371.60	< 0.0001
A-C	${3.73}\times{10}^{-7}$	1	${3.73}\times{10}^{-7}$	35.41	0.0006
B-Q	0.0001	1	0.0001	6589.32	< 0.0001
AB	${2.78}\times{10}^{-8}$	1	${2.78}\times{10}^{-8}$	2.64	0.1483
A^2^	${5.31}\times{10}^{-7}$	1	${5.31}\times{10}^{-7}$	50.46	0.0002
B^2^	${9.91}\times{10}^{-7}$	1	${9.91}\times{10}^{-7}$	94.20	< 0.0001
Residual	${7.36}\times{10}^{-8}$	7	${1.05}\times{10}^{-8}$		
Cor total	0.0001	12			

*df* degree of freedom.

**Table 5:** Fit statistics table for *f*.

Std. Dev	0.0001	*R*^2^	0.999
Mean	0.0325	Adjusted *R*^2^	0.998
C.V. %	0.3158	Predicted *R*^2^	0.989
		Adeq Precision	106.23

### Assumption of normal distribution

**Figure 5:**
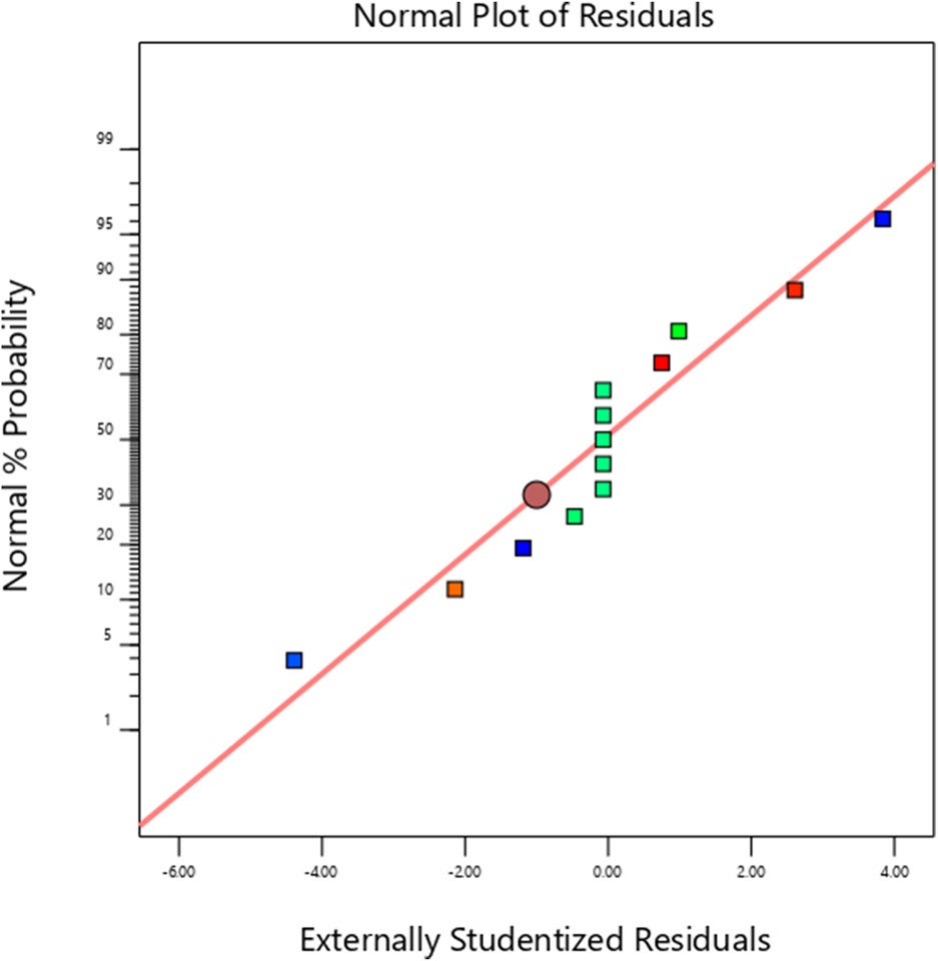
Normal plot of residuals.

In a normal probability plot of residuals (Fig. 5), if the data points fall on a straight line, the residuals follow a normal distribution. If the data points form an S-shaped curve, the data does not follow a normal distribution, and the mathematical model should be modified. From the normal probability plots of the experimental data, it can be seen that the residuals are distributed on both sides of the straight line and the data points are close to the line, indicating that the residuals of the experimental data follow the normal distribution assumption.

### Constant variance assumption

**Figure 6:**
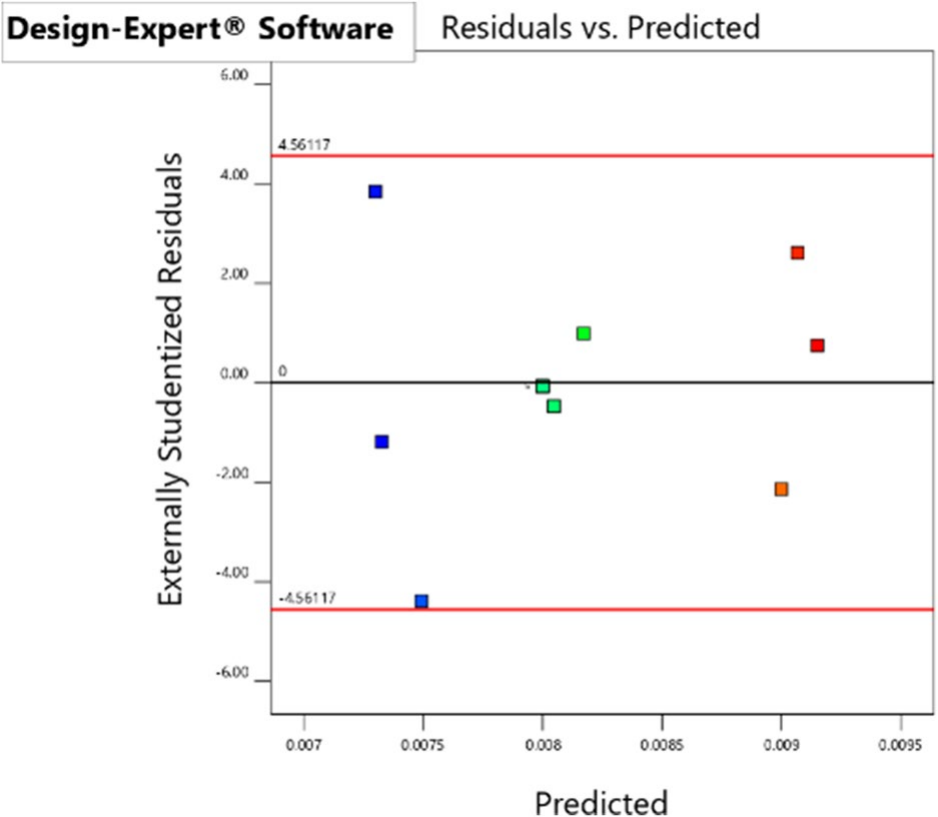
Residuals versus predicted coefficient of Darcy coefficient of friction.

The assumption of constant variance can be tested by examining the relationship between the predicted values and the residuals. If the residuals are constrained within a certain range, then the second assumption of the variance analysis (constant variance) is satisfied; if the residuals increase with the increase of the predicted values, then this assumption is not satisfied, and the model should be modified. From the distribution of the friction factor residuals and predicted coefficients in Fig. 6, it can be seen that the experimental residuals are evenly distributed within the range of ± 4.56, and there is no diverging trend in the experimental value variance as the predicted values increase. Therefore, the experimental values satisfy the assumption of constant variance.

### Assumption of randomness

**Figure 7:**
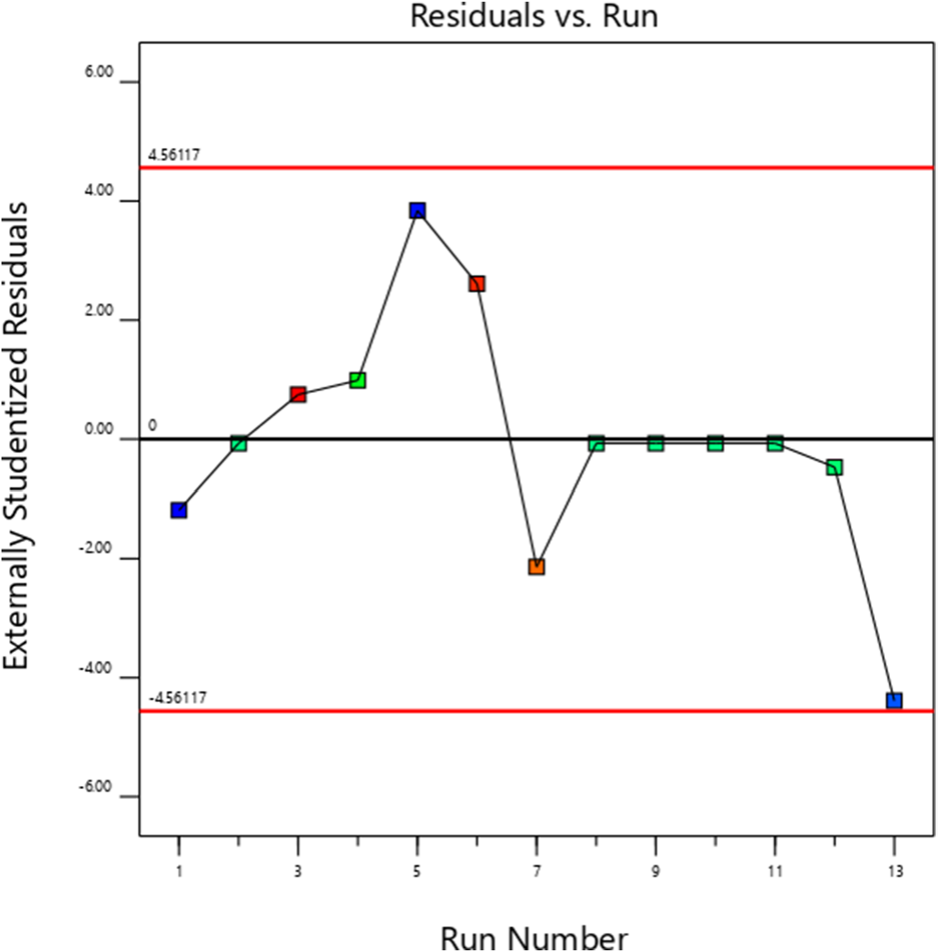
Run number versus residuals.

The randomness hypothesis can be tested by examining the correlation between the sequence of runs and the residuals. If there is a clear time-related trend in the relationship between the sequence of runs and the residuals, it indicates that the response involves time-related variables, and the experimental design needs to be modified; otherwise, the data is randomly distributed. Figure 7 shows that the residual data is within the range of ± 4.56, and there is a clear randomness in the relationship between the running time and the residual data. Therefore, the variables do not involve time-relatedness and meet the randomness hypothesis.

Based on the above analysis, the test data meet the three basic assumptions, which proves that the test method and test data meet the requirements.

*P*-value is a statistical term used to measure the significance of a hypothesis. In RSM, analysis of variance (ANOVA) is usually performed to calculate the P-value of each factor. If the P-value is less than 0.05, it indicates that the factor's influence on the response variable is significant and can be considered as an important factor. If the *P*-value is greater than 0.05, it indicates that the factor's influence on the response variable is not significant and can be ignored. Equation (11) considers all actual factors and is apparently lengthy and complicated. Several factors listed in the ANOVA table, i.e. ${{CQ}}$, have *P* values greater than 0.05, indicating that these factors are not significant and therefore can be excluded from the ANOVA table to simplify the model. After this simplification, the ANOVA table is shown in Table 4. The simplified correlation is shown in Eq. (12).

12 $$f = 2.24 \times 10^{ - 2} - 1.06 \times 10^{ - 4} C - 7.2 \times 10^{ - 5} Q + 1.10 \times 10^{ - 6} C^{2} + 9.36 \times 10^{ - 8} Q^{2}$$

The analysis process above is based on the three ANOVA assumptions of the RSM analysis method (normality of data, constant variance, and randomness). Below is the verification process for these three assumptions.

**Figure 8:**
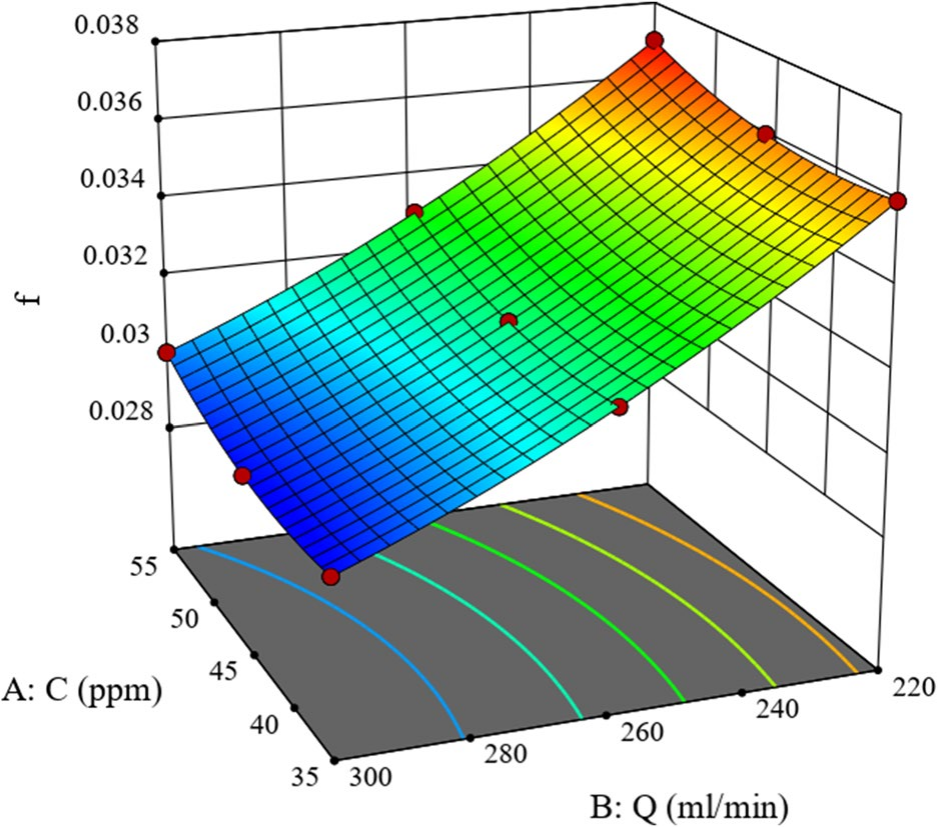
Response surface curve of friction factor versus *Q* and *C*.

**Figure 9:**
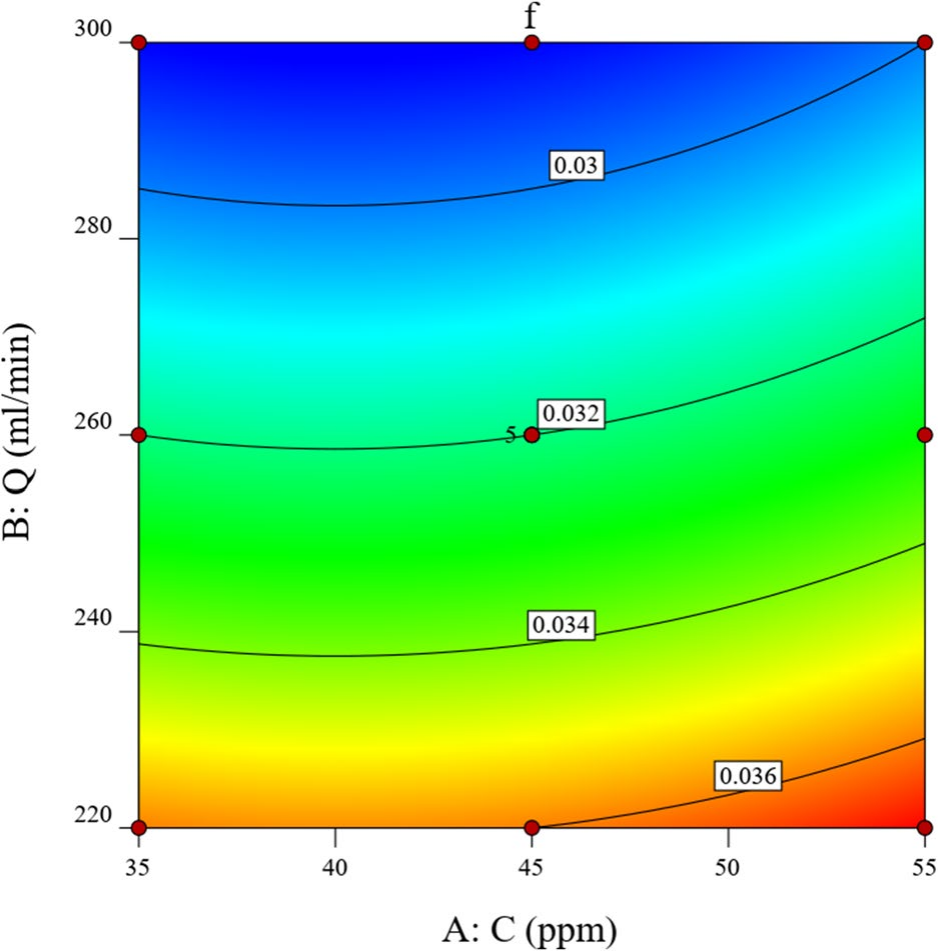
Control plot of friction factor versus *Q* and *C*.

The friction coefficient of the drag-reducing solution can be well predicted by Eq. (12) in the variance analysis of the RSM model. Under experimental conditions, i.e., a drag-reducing agent concentration of 35–55 ppm and a volumetric flow rate of 220-300 ml/min, when the first-order coefficient of the drag-reducing agent concentration *C* and the volumetric flow rate *Q* in the optimized formula are both negative, and the second-order coefficients are both positive. It can be seen that when the values of *C(Q)* remain unchanged, *f* and *Q(C)* satisfy a quadratic function relationship, the intercept is a positive value, and the axis of symmetry is to the right of the *f* axis. Therefore, there exists extreme points in the positive area of *Q(C)*. This trend is clearly shown in Figs. 8 and 9. It is evident from the data that *f* consistently exhibits a downward trend as the values of both *C* and *Q* decrease (or increase), without any instances of an upward change within this range. Consequently, it can be inferred that the minimum value of *f* does not fall within the scope of these conditions.

**Figure 10:**
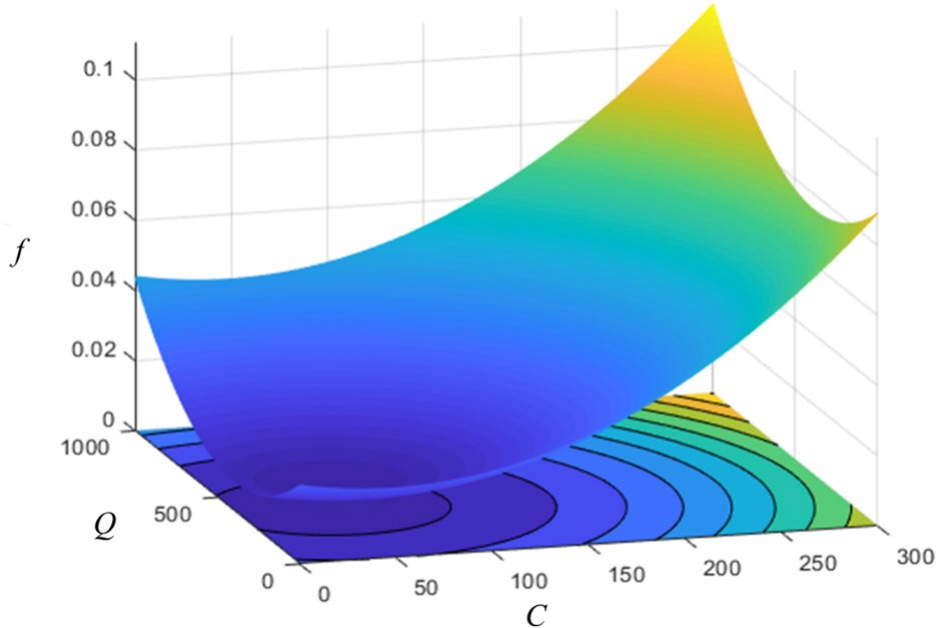
Response surface of friction factor versus *Q* and *C*.

To verify the above analysis, the range of *C* and *Q* in Eq. (12) was expanded to 0–300 ppm and 0–1000 ml/min, and Fig. 10 was obtained. It can be determined from the figure that *f* has a minimum value of 0.005987 (*C* = 48 ppm, *Q* = 384 ml/min), which corresponds to the analysis process. In reference 41, the polymer drag-reducing agent concentration *C* was linearly increased from 0 to 80 ppm at a constant Reynolds number *Re* of 5200, and it was found that the friction coefficient *f* continued to decrease, approaching the maximum drag reduction asymptote when *C* = 48 ppm^41^. The concentration calculated by the correlation formula is the same, and the Reynolds number at this time is 4820, similar to the Reynolds number in the literature. This difference may be caused by the pipe diameter effect.

**Figure 11:**
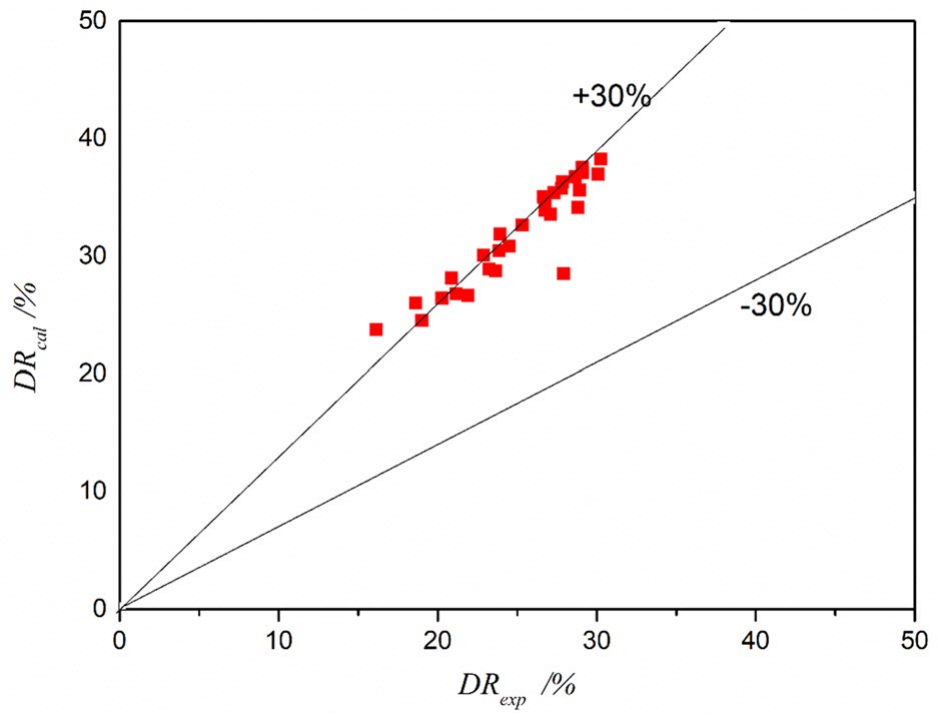
Interpolated values by the correlation versus actual values.

We utilized Eq. (12) to interpolate data points and derive values for other points within a specified range. Specifically, within the range of drag reducer concentrations of 35–55 ppm and volume flow rates of 220–300 ml/min, we established a correlation between the experimental data and the data predicted by the formula. The results of this correlation are illustrated in Fig. 11. It is evident from Fig. 11 that the majority of the data points fall within a 30% margin and are positioned closer to the upper limit of this range. This observation underscores a strong correlation between the experimental data and the predictions derived from the formula.

**Figure 12:**
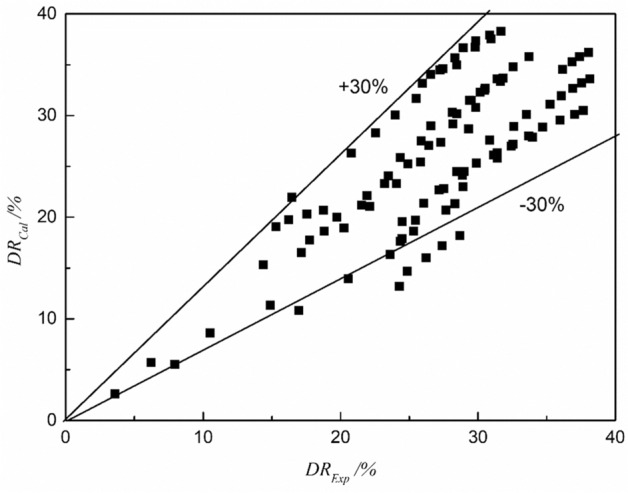
Extrapolation values by the correlation versus actual values.

Data extrapolation refers to the derivation of new data points beyond the range known data points. Its applications include prediction or estimation of data values. Unlike data interpolation, data extrapolation requires global approximation of functions from known data points to derive values beyond the given data range. We expand the experimental range to a larger range outside the range of drag reducer concentration of 35–55 ppm and volume flow of 220–300 ml/min (170 < *Q* < 350 ml/min, 15 < *C* < 35 ppm; 170 < *Q* < 350 ml/min, 55 < *C* < 95 ppm;170 < *Q* < 220 ml/min, 35 < *C* < 55 ppm; 300 < *Q* < 350 ml/min, 35 < *C* < 55 ppm) to verify the accuracy of Eq. (12), see Fig. 12. It can be clearly seen from Fig. 12 that most of the data points are concentrated in the effective range of ± 30%. Compared with the interpolated data, the uniformity of the data distribution is better, which proves that the experimental data in the range outside Table 3 also has good correlation with forecast data. Under this condition, we can confirm that our correlation Eq. (12) can be used within and without the range of data provided by our microflow device.

## Conclusion

In this study, we investigate the drag reduction property of slurry in microtube flow using response surface methodology. First, we manifest our measured data by microtube-flow device are correct and explain why some phenomena are different from the ones in classic theory; second, we show that our new drag-reducing agent can reduce the resistance in microtube flow under different concentrations; third, we further use RSM to establish a correlation between drag reduction and operation condition in microtube flow (flowrate and drag-reducing agent concentration) to predict the drag reduction. Results show that our correlation works well; we indeed use more data to validate the correctness of our correlation, and results are satisfying. Hence, we can substantiate that this novel drag-reducing agent is amenable to quantitative analysis, thereby enabling the development of a predictive model for its implementation in industrial systems, including but not limited to Heating, Ventilation, and Air Conditioning (HVAC) systems.

## Data Availability

To datasets generated and analyzed during the current study are not publicly available due to the policy of Shandong Institute of Petroleum and Chemical Technology, but are available from the corresponding author upon reasonable request.

## List of symbols

*C*: Drag reducer concentration (ppm)
*Q*: Volume flow (ml/min)
Δ*p*: Pressure drop (Pa)
*f*: Darcy friction coefficient (–)
$\rho$: Density of the solution (kg m^−^^3^)
*U*_*b*_: Mean velocity (m/s)
*D*: Inner diameter (m)
*r*: Inner radius (m)
*l*: Tube length (m)
*DR*: Drag reduction rate (–)
$$\mu$$: Viscosity(mPa s)
*Re*: Reynolds number (–)

## Subscripts

ANOVA: Analysis of variance
*DRP*: Drag reducing polymer
*CRD*: Completely Randomized Design
*CCD*: Central Composite Design
*s*: Solution without polymer
*p*: Solution with polymer
